# Clinical correlates of a high cardiorespiratory risk score for very low birth weight infants

**DOI:** 10.1038/s41390-024-03580-y

**Published:** 2024-09-19

**Authors:** Sherry L. Kausch, Claire C. Slevin, Amanda Duncan, Karen D. Fairchild, Douglas E. Lake, Jessica Keim-Malpass, Zachary A. Vesoulis, Brynne A. Sullivan

**Affiliations:** 1 Department of Pediatrics, Division of Neonatology, University of Virginia School of Medicine, Charlottesville, VA USA; 2 Department of Pediatrics, Johns Hopkins University, Baltimore, MD USA; 3 Department of Pediatrics, Division of Newborn Medicine, Washington University in St. Louis, St. Louis, MO USA; 4 Department of Medicine, Division of Cardiology, University of Virginia School of Medicine, Charlottesville, VA USA; 5 Department of Pediatrics, Division of Hematology, University of Virginia School of Medicine, Charlottesville, VA USA

## Abstract

**Background:**

A pulse oximetry warning system (POWS) to analyze heart rate and oxygen saturation data and predict risk of sepsis was developed for very low birth weight (VLBW) infants.

**Methods:**

We determined the clinical correlates and positive predictive value (PPV) of a high POWS score in VLBW infants. In a two-NICU retrospective study, we identified times when POWS increased above 6 (POWS spike). We selected an equal number of control times, matched for gestational and chronologic age. We reviewed records for infection and non-infection events around POWS spikes and control times. We calculated the frequencies and PPV of a POWS spike for infection or another significant event.

**Results:**

We reviewed 111 POWS spike times and 111 control times. Days near POWS spikes were more likely to have clinical events than control days (77% vs 50%). A POWS spike had 52% PPV for suspected or confirmed infection and 77% for any clinically significant event. Respiratory deterioration occurred near more POWS spike times than control times (34% vs 18%).

**Conclusions:**

In a retrospective cohort, infection and respiratory deterioration were common clinical correlations of a POWS spike. POWS had a high PPV for significant clinical events with or without infection.

**Impact:**

- There are significant gaps in understanding the best approach to implementing continuous sepsis prediction models so that clinicians can best respond to early signals of deterioration.
- Infection and respiratory deterioration were common clinical events identified at the time of a high predictive model score.
- Understanding the clinical correlates of a high-risk early warning score will inform future implementation efforts.

## Introduction

Late-onset sepsis is a life-threatening condition that occurs in up to 15% of premature infants with very low birth weight (VBLW, <1500 g) and is characterized by a systemic inflammatory response to infection that often includes changes in heart rate and oxygenation patterns.^1–3^ Early detection of sepsis can allow for early initiation of antibiotics and other interventions to prevent disease progression.^4^ We previously developed and externally validated the Pulse Oximetry Warning Score (POWS), a predictive model that detects abnormal patterns in continuous HR and SpO_2_ data during the early phase of sepsis. In this prior work, we trained the POWS model to predict the risk of blood culture-positive late-onset sepsis in the next 24 h.^5^ We then evaluated the performance of the model to predict sepsis across three NICUs. POWS performed well, having good calibration and with area under the receiver operator characteristic curve (AUC) ranging from 0.782 to 0.827 across sites.

While these AUC’s indicate good discrimination between time with and without confirmed sepsis, rare events inevitably lead to some false positive predictions. In the case of late-onset sepsis, the average risk in any 24 h period is 0.25%. The systemic inflammatory response to sepsis causes autonomic dysfunction leading to the cardiorespiratory instability and altered vital signs detected by POWS,^6,7^ but this pathophysiology is not specific to bloodstream infection. Blood culture-negative infections and respiratory diseases not related to infection can cause similar physiological alterations due to systemic inflammation.^8^ Thus, while POWS was developed and validated for predicting late-onset sepsis with bacteremia, we hypothesized that acute rises in POWS would also correspond with events of cardiorespiratory deterioration due to conditions other than confirmed sepsis.^6,8,9^

In this study, we sought to understand the clinical context around the time of a high POWS score. We used a two-NICU retrospective cohort to quantify the occurrence of documented clinical events near an acute rise or “spike” in POWS over the prior baseline in preterm VLBW infants as compared to times without a rise in POWS. Our goal was to understand the clinical associations of a significant rise in the predicted risk.

## Methods

### Patient population and study design

We conducted a retrospective cohort analysis using data from two-level IV academic NICUs (University of Virginia Children’s Hospital and Washington University/St. Louis Children’s Hospital). The study was approved at each institution under a waiver of consent by their respective IRBs. At each center, heart rate (HR) and oxygen saturation (SpO_2_) are recorded every one or two seconds from the bedside monitor and archived from NICU admission through discharge. In the current study, we excluded VLBW infants with major congenital or chromosomal anomalies, as many have abnormal cardiorespiratory patterns at baseline. All remaining VLBW infants for whom archived HR and SpO_2_ data were available to calculate POWS were included. Missing HR and SpO2 data occurred at random for various technical reasons. Infants who died within 3 days of a POWS spike were excluded.

### POWS calculation and spikes

We calculated model input features (mean, standard deviation, skewness, kurtosis, and cross-correlation of HR and SpO_2_) every 10 min from the raw monitoring data. We used the calculated features to obtain hourly POWS risk predictions from 3 days of age to hospital discharge for all infants where data were available.^5^ We excluded the first 3 days of data to eliminate high POWS scores related to early-onset sepsis or transitional physiology. The POWS value represents the hourly fold-increased risk of late-onset sepsis diagnosis in the next 24 h compared to the baseline sepsis risk for all VLBW infants in our cohort at all times. For example, a POWS value of 3 indicates an infant has a 3-fold increased risk of imminent sepsis diagnosis compared to the baseline risk at any time for a VLBW infant. This POWS value is based on the model detecting abnormal HR and SpO_2_patterns.

We evaluated a range of high POWS thresholds to define a spike by analyzing the resulting frequency and sensitivity for blood culture-positive sepsis. We selected a high threshold at a POWS of 6, A POWS threshold of 6 represents only 1.9% of observed scores and is associated with a 58% sensitivity for culture-positive sepsis. A lower threshold would have higher sensitivity, but we focused on an extremely high-risk value to narrow our analysis to the most significant events. We further defined POWS spikes as rising above the threshold of 6 after remaining below 6 for the previous 5 days. All POWS spikes were identified, and a random sample, allowing one per infant, was selected for chart review. Every POWS spike date and time was matched with a control date and time at equal gestational and postnatal ages whose POWS score near that time did not surpass the threshold. When infants could not be matched by both GA and postnatal age, we matched by GA and selected a control timestamp at the nearest postnatal age with POWS < 6. Infants with POWS spikes were not excluded from control matching, so infants with a POWS spike could also have a control time matched with a POWS spike in another infant, as long it occurred 10 days or more from the POWS spike time.

### Chart review

A list of dates and times corresponding to POWS spikes or control times was provided to reviewers at each site who were not involved in the statistical analysis and were blinded to the spike type (POWS spike or control time). Reviewers were instructed to record any significant event documented in the medical record within 3 days, defined as the day of the timestamp, −2 calendar days, +2 calendar days. We included days before and after the timestamp because the timing of clinical diagnosis is variable and cannot be determined precisely from chart review. Events could include a change in patient status, change in management, new diagnosis, or significant abnormality in laboratory or radiography results. Data sources included progress notes, problem lists, radiographs, operative notes, nursing and respiratory care flowsheets, and laboratory results during the five days centered on each POWS spike or control time. A second blinded reviewer at each site audited the first reviewer’s work for clarifications and questions.

Together, the two reviewers (still blinded) determined the primary clinical correlation with the spike or control time as falling into one of nine pre-defined infection-related and non-infection-related categories (Table 1). For times when more than one clinical correlation occurred, the primary clinical correlation was designated as infection-related over non-infection-related categories if one was present. For example, when respiratory deterioration prompted blood culture and sepsis diagnosis, the primary clinical correlation was categorized as sepsis and not respiratory deterioration. Of note, POWS scores were calculated for research purposes after infants were discharged from the NICU and thus could not have impacted clinical care.

**Table 1:** Definitions of clinical diagnoses assigned after chart review.

Diagnosis	Definition
1. Confirmed late-onset sepsis	Signs of sepsis and a positive blood culture at >72 h of age treated with antibiotics for at least 5 days
2. Clinical sepsis or other infection	A negative blood culture treated with at least 5 days antibiotics due to high clinical suspicion, including urinary tract infections and pneumonia
3. Sepsis ruled out	Negative cultures, <5 days antibiotics
4. Necrotizing enterocolitis or intestinal perforation	Clinical signs and imaging showing pneumatosis intestinalis, portal venous air, or pneumoperitoneum and at least 5 days of treatment
5. Surgery or procedure	Requiring anesthesia or sedation
6. Respiratory deterioration	Moving to a higher mode of respiratory support or a substantial increase in ventilator settings due to respiratory acidosis or hypoxemia
7. Increased apnea	A clinically significant increase in apnea frequency and severity
8. Other significant event	A documented change in clinical condition that does not fall into any other category
9. No event	No documented change or diagnosis in the defined time period

### Statistical analysis

We used summary statistics to analyze cohort characteristics. Frequencies of the categories of primary clinical correlations assigned for POWS spikes and control times were compared using Fisher’s exact test. We also analyzed clinical correlations of POWS spikes and control times according to whether there was a blood culture for suspected or confirmed sepsis and whether there was respiratory deterioration, taking events with both types of clinical correlation into consideration. We calculated the positive predictive value of POWS spikes to detect A) suspected or confirmed infection, B) respiratory deterioration, and C) any clinically significant event.

## Results

### Patients and POWS spikes

We analyzed data from 806 VLBW infants, 50% male, with a mean gestational age of 28.0 ± 2.8 weeks and mean birth weight of 997 ± 293 g. Of these, 285 infants (35%) had one or more hourly POWS values that met the criteria for a spike during their NICU stay (Table 2). The relative frequency of POWS scores > 6 constitutes approximately 2% of all recorded values. In this cohort, 475 POWS spikes were present in 30,666 patient days of data. To give a sense of the spike frequency, in a NICU with an average daily census of 20 VLBW infants, this would equate to one spike every 3.2 days.

**Table 2:** Characteristics of infants included in medical record review of POWS spike or control times.

Characteristic	Control *N* = 111	POWS Spike *N* = 111	*p*-value^a^
Gestational age (weeks)	26 (2.0)	26 (2.0)	>0.9
Birth weight (grams)	939 (245)	867 (233)	0.036
Male sex	61 (55%)	63 (57%)	0.8
Race			0.2
Black	37 (33%)	46 (41%)	
White	68 (61%)	63 (57%)	
Other/Unknown	6 (5%)	2 (2%)	
Age at alert (days)	20 (25)	17 (18)	0.6

Values are either mean ± standard deviation or number and percentage.

^a^*P*-values were calculated using Wilcoxon rank sum tests for continuous variables or chi-square goodness of fit test for proportions.

### Clinical associations of POWS spikes

**Fig. 1:**
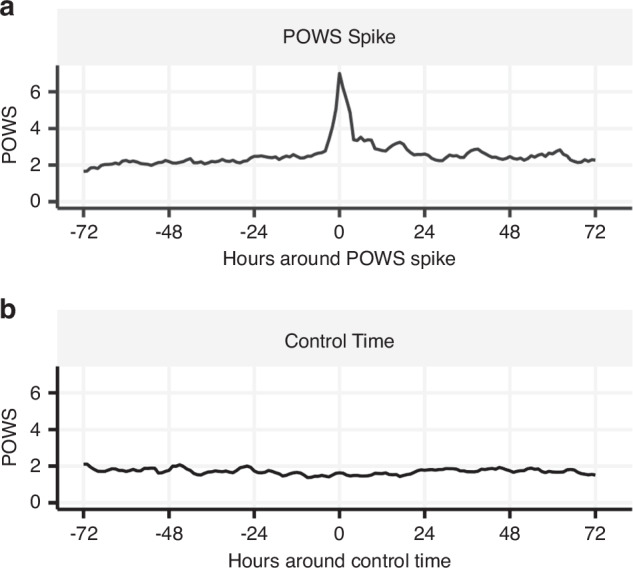
Average POWS scores around POWS spikes and control times. Plots of the average hourly POWS scores from 3 days before to 3 days after a (**a**) POWS spike and (**b**) control time matched for gestational age and postnatal age.

**Fig. 2:**
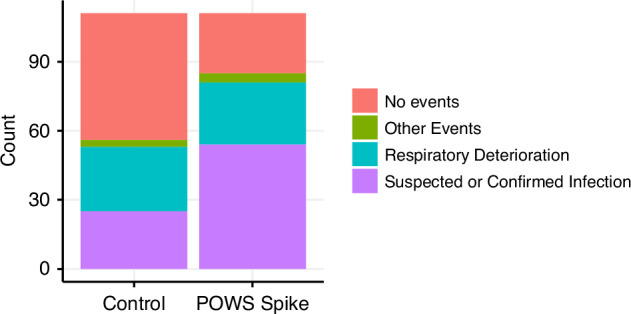
Clinical associations with POWS spikes and matched control times. Clinical conditions defined in Table 3 were grouped into the major categories of suspected or culture-confirmed infection, respiratory deterioration (including increased apnea), and other clinical events.

We randomly selected 111 POWS spike times and matched them with 111 control times from 192 VLBW infants (Table 2). Fig. 1a shows the average POWS scores around the time of a spike for infants in the POWS spike group. Fig. 1b displays the average POWS scores for the matched controls. Infants with POWS spikes had similar sex, race, and ethnicity as infants selected as controls, but they had slightly lower mean birthweight despite matching for gestational age (Table 2). Postnatal age at the timestamp used for chart review was also similar between the two groups (17 days old at POWS spikes vs 20 days old at control times, Table 2). The average POWS score was 7.0 (SD = 1.6) at spike times and 1.3 (SD = 0.9) at control times. Clinically significant events were significantly more likely to occur within 3 days of POWS spike times compared to control times (spike times 85/111 (77%) with identified events; control times 56/111 (50%) with identified events (*p* < 0.001, Fig. 2). Table 3 shows the frequencies of the primary clinical correlation identified for POWS spikes and control times. The PPV of a POWS spike was 52% for suspected or confirmed sepsis, 34% for respiratory deterioration, and 77% for any significant clinical event.

**Table 3:** Clinical correlations of POWS spike times and matched control times.

Primary event	Control *N* = 111	POWS Spike *N* = 111
Confirmed late-onset sepsis^a^	3 (2.7%)	10 (9.0%)
Clinical sepsis or other infection	9 (8.1%)	13 (12%)
Sepsis ruled out	12 (11%)	20 (18%)
Necrotizing enterocolitis or intestinal perforation^a^	1 (0.9%)	11 (9.9%)
Surgery or procedure	2 (1.8%)	3 (2.7%)
Respiratory deterioration	12 (11%)	16 (14%)
Increased apnea	16 (14%)	11 (9.9%)
Other significant events	1 (0.9%)	1 (0.9%)
No events^a^	55 (50%)	26 23%)

Values are numbers and percentages. The categories for Respiratory deterioration and increased apnea report numbers of POWS spike and control times where this was the primary clinical correlation and no evaluation was performed for suspected or confirmed infection. The “other significant event category” included one event in each category where hypotension requiring treatment was documented, but no evaluation was performed for suspected infection or respiratory deterioration.

^a^*p* < 0.05 by Fisher’s exact tests.

An evaluation for suspected sepsis or NEC occurred within 3 days of a higher proportion of POWS spikes than control times (58/111 (52%) vs. 27/111 (24%), *p* < 0.001). Of the 58 POWS spikes identified close in time to blood cultures for suspected sepsis or NEC, 32 (55%) preceded the blood culture order with a median lead time of 18.5 h (IQR 13–41 h). Respiratory deterioration, with or without suspected infection, also occurred near a higher proportion of POWS spikes than control times (38 (34%) vs. 20 (18%), *p* = 0.009). Respiratory deterioration or increased apnea without suspected infection occurred at similar frequencies (POWS spikes, 27 (24%) vs. control times, 28 (25%)). Of the 38 POWS spikes near respiratory deterioration or increased apnea, 28 (82%) of the spikes occurred on or before the day of the clinical diagnosis.

## Discussion

We quantified clinical events near a rise in POWS above a high threshold to understand the clinical context and, thus, how the risk score might inform decision-making. Infection and respiratory deterioration were the most common clinical events identified at the time of high POWS scores and occurred significantly more often near POWS spikes than control times. Considering the rare incidence of cardiorespiratory deterioration throughout the NICU hospitalization in VLBW infants, a POWS spike had a relatively high PPV for infection, respiratory deterioration, and other significant clinical events.

This work draws from our experience with heart rate characteristics (HRC) monitoring for late-onset sepsis in VLBW infants.^8,10,11^ Both POWS and HRC monitoring use physiologic data and predictive modeling for early warning of sepsis, but POWS incorporates SpO_2_ analytics with HRC, better capturing the cardiorespiratory deterioration associated with sepsis and sepsis-like syndromes. The POWS algorithm also includes the cross-correlation of HR and SpO_2_, which increases when an infant is experiencing increased apnea, HR decelerations, and oxygen desaturation.^6,12^ Respiratory deterioration and increases in apnea are common early signs of infection.^9^ However, such events also occur in premature infants without infection due to lung disease and immature respiratory control.^13^

Abnormal cardiorespiratory patterns occur during neonatal sepsis due to the autonomic response to systemic inflammation. Afferent parasympathetic nerves are stimulated by pathogen toxins and inflammatory molecules in the bloodstream or tissues and relay signals to the brainstem, triggering a cholinergic anti-inflammatory response, which is a critical host defense mechanism.^14^ Efferent vagus nerve signals act on muscarinic cholinergic receptors on leukocytes to decrease proinflammatory cytokine production. Vagus nerve firing at the sinoatrial node triggers transient heart rate decelerations.^15^ Cytokines and prostaglandin-E2 bind to receptors in the brainstem that control respiratory rhythmogenesis, resulting in apnea, which is often accompanied by bradycardia and desaturation.^7,16,17^ POWS uses features that detect patterns of decreased heart rate variability, increased bradycardia events, increased intermittent hypoxemia, and increased apnea events.^5^ While these physiologic disturbances are frequent signs of sepsis, they are non-specific. Non-infectious conditions in preterm infants, such as respiratory distress syndrome and chronic lung disease, are associated with inflammation and cardiorespiratory instability.^18^ The results of this study suggest that while infection is more common when POWS is high, respiratory deterioration without infection can be included in the differential diagnosis when evaluating a patient with a significant rise in the score over their prior baseline. Thus, while developed for sepsis, knowing that POWS can provide early warning of cardiorespiratory deterioration with or without sepsis may broaden the clinical utility of predictive monitoring in the NICU.

A limitation of our approach is the relatively small sample size of POWS spike time periods reviewed but a strength is the careful medical record review of the 222 spike and control times by two investigators and the inclusion of two NICUs. The retrospective nature of the study limited the ability to identify undocumented clinical events. However, we note that events documented in daily progress notes are generally more likely to be clinically significant, and other corroborating sources in the medical record, such as flowsheets, laboratory test results, radiographs, and nursing notes were also reviewed. Despite matching control times to have similar gestational and postnatal age as POWS spikes, the mean birth weight of the infants with POWS spikes was lower by ~70 g, a magnitude unlikely to have a significant impact on the analysis. The limitations of this study will inform prospective analysis, which will be necessary to determine the clinical impact and utility of such a system.

We focused the current analysis on a high threshold occurring at low frequency to capture the clinical context of infrequent but extremely high-risk POWS scores. Our intention was not to analyze the sensitivity and specificity of a specific alert threshold for implementation in the clinical setting, as this would have required a detailed analysis of every clinically significant event throughout the NICU course. Importantly, a POWS threshold lower than 6 would yield higher sensitivity for infection or respiratory deterioration and may still occur at a clinically reasonable frequency that would not contribute to alarm fatigue.^19,20^

A predictive monitoring system without threshold-based alerts avoids the issue of alarm fatigue and false positives but requires users to be proactive rather than reactive in evaluating the scores and interpreting the information.^21,22^ On the other hand, implementing a risk score with alert thresholds can trigger clinical response guidelines intended to prompt action at a discrete level of predictive accuracy. Our analysis of a clinical prediction model using in-depth chart review and matched controls is unique and provides a foundation for implementation in a clinical trial setting. Further evaluation is necessary to understand the impact POWS will have on clinical care, including risks and benefits.

## Conclusions

In VLBW infants at 2 NICUs, a spike in the predicted risk of late-onset sepsis using POWS, a continuous model based on cardiorespiratory data was associated with infection, respiratory deterioration, and other significant clinical events. A POWS spike had a 52% PPV for suspected or confirmed infection and 77% PPV for any clinically significant event. These results will inform the next steps towards implementation of POWS, such as prospective pre-implementation evaluation and clinical trials.

## Data Availability

The datasets generated during and/or analyzed during the current study are available from the corresponding author on reasonable request.
